# As fast as an X-ray: real-time magnetic resonance imaging for diagnosis of idiopathic scoliosis in children and adolescents

**DOI:** 10.1007/s00247-024-05919-3

**Published:** 2024-04-30

**Authors:** Christian Roth, Christoph-Eckhard Heyde, Eckehard Schumann, Dirk Voit, Jens Frahm, Franz W. Hirsch, Rebecca Anders, Daniel Gräfe

**Affiliations:** 1 Institut für Kinderradiologie, Liebigstr. 20a, 04103, Leipzig, Germany; 2 Department of Orthopedic and Trauma Surgery, Spine Department, University of Leipzig, Leipzig, Germany; 3 Biomedizinische NMR, Max-Planck-Institut Für Multidisziplinäre Naturwissenschaften, Göttingen, Germany

**Keywords:** Child, Magnetic resonance imaging, Scoliosis, Supine position

## Abstract

**Background:**

Idiopathic scoliosis is common in adolescence. Due to the rapid growth of the spine, it must be monitored closely with radiographs to ensure timely intervention when therapy is needed. As these radiographs continue into young adulthood, patients are repeatedly exposed to ionizing radiation.

**Objective:**

This study aimed to investigate whether real-time magnetic resonance imaging (MRI) is equivalent to conventional radiography in juvenile idiopathic scoliosis for determining curvature, rotation and the Risser stage. Additionally, the time requirement should be quantified.

**Materials and methods:**

Children with idiopathic scoliosis who had postero-anterior whole-spine radiography for clinical indications were included in this prospective study. A real-time spine MRI was performed at 3 tesla in the supine position, capturing images in both the coronal and sagittal planes. The scoliosis was assessed using Cobb angle, rotation was evaluated based on Nash and Moe criteria, and the Risser stage was determined for each modality. The correlations between modalities and a correction factor for the Cobb angle between the standing and supine position were calculated.

**Results:**

A total of 33 children (aged 5–17 years), who met the inclusion criteria, were recruited. The Cobb angle (*R*^2^ = 0.972; *P* < 0.01) was positively correlated with a correction factor of 1.07 between modalities. Additionally, the degree of rotation (*R*^2^ = 0.92; *P* < 0.01) and the Risser stage (*R*^2^ = 0.93; *P* < 0.01) demonstrated a strong correlation.

**Conclusion:**

Real-time MRI is equivalent to conventional radiography in determining baseline parameters. Furthermore, it is radiation-free and less time-consuming.

**Graphical abstract:**

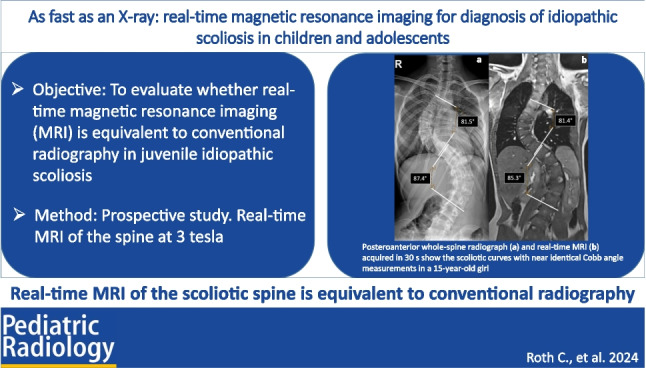

## Introduction

Idiopathic scoliosis is common in adolescents, with a prevalence of up to 5%, of which about 20% require treatment [1]. Due to the rapid growth of the spine in this age group [2], the development of scoliosis must be regularly documented by radiographs of the spine in the posteroanterior (PA) and sagittal planes. The therapeutic consequences must be considered, based on the type and severity of the scoliosis [1]. The parameters used to define severity include the scoliosis angle as per the Cobb method [3], rotation based on Nash and Moe’s approach [4], and skeletal maturation in accordance with the Risser stage [5].

Delayed therapy can impair lung development, leading to pulmonary hypertension [6]. Furthermore, changes in body dynamics may lead to muscular imbalances, chronic pain, and cosmetic problems [7]. These possible consequences highlight the importance of early diagnosis and thorough monitoring. Conventional full-spine radiography has been the gold standard in scoliosis diagnosis due to its widespread availability, low cost, and straightforward image interpretation. Measurement methods are based on conventional radiography [8] or on the EOS system (EOS® Imaging, Paris, France) [9]. However, the repetitive exposure of the thorax and abdomen to ionizing radiation is a disadvantage. Thus, an increased incidence of mammary and endometrial carcinomas is reported after many years of repeated X-ray examinations for scoliosis [1]. Authors have estimated the lifetime risk of cancer in girls to be twice as high as that in boys [10, 11].

Radiation-free alternatives, such as sonographic analysis, can only partially assess the condition [12]. Magnetic resonance imaging (MRI) has been identified as an equivalent substitute for Cobb angle assessment [8, 13, 14]. Variations in Cobb angle measurements may arise due to differences in body positioning during imaging modalities, such as lying down for MRI and standing upright for X-ray. Correction factors specific to scoliosis have been documented to account for these discrepancies [8, 13]. These factors eliminate the need for a compressorium in the supine position [14].

**Fig. 1:**
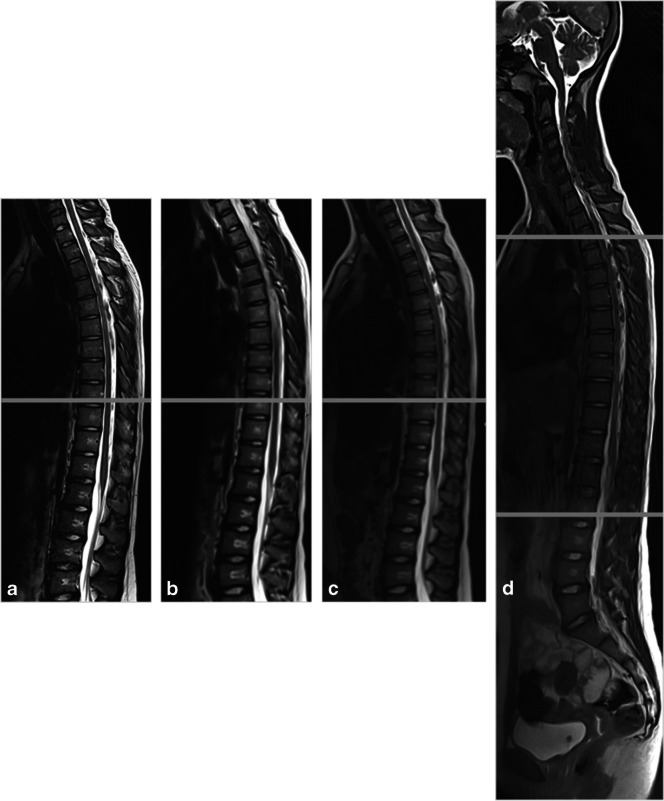
Sagittal spine acquisition time of real-time and conventional magnetic resonance imaging. A 13-year-old girl with a history of lower back pain and suspicion of scoliosis. Compared to the normal T2-sequence (5:15 min) (**a**), the fast T2-sequence (1:42 min) (**b**) and the half-Fourier-acquisition single-shot turbo spin echo (1:28 min) (**c**) with two stacks each, the real-time magnetic resonance imaging sequences (**d**) are faster (0:38 min), even with three stacks. The boundaries of vertebral bodies can be detected

Unfortunately, MRI is less commonly available, more expensive, and requires an examination time of 20–60 min [13, 15] during which the patient must not move excessively. This situation can be challenging for younger children, but a recent study has shown that even in such cases, MRI can reveal relevant findings [16]. A novel, fast low-angle shot MRI technique (FLASH 2.0) can now provide a radiation-free, ultra-fast alternative to radiography suitable for daily use. With up to 50 frames per second, FLASH 2.0 real-time MRI is largely unaffected by motion [17–19]. Three stacks covering a field of view from the posterior fossa to the greater trochanter can be acquired within 30 s. The loss of image quality in depecting the vertebral endplates when obtaining the measurements is acceptable. Figure 1 compares real-time MRI of the whole spine in the sagittal plane with commonly available T2-weighted sequences and their respective time-requirements.

In this study we aimed to investigate whether the new FLASH 2.0 real-time magnetic resonance imaging real-time (MRI) is equivalent to radiography in diagnosing juvenile idiopathic scoliosis in children and adolescents, specifically in terms of the Cobb angle [3], rotation (according to Nash and Moe [4]), and Risser stage [5] parameters. The secondary objectives were to quantify the time required for real-time MRI compared with radiography and establish a correction factor for the Cobb angle between real-time MRI and conventional radiography.

## Materials and methods

Before commencing the study, the local ethics committee granted a favorable opinion. Written informed consent was obtained from the participants or their legal representatives. This was a prospective, monocentric study. Patients aged 5–17 years with idiopathic scoliosis who had PA and lateral erect spine radiographs as part of regular diagnostics at the pediatric orthopedic outpatient clinic between January 2021 and November 2022 were included. As it is known that scoliosis may increase by up to 5° during the day [20], conventional radiography and MRI were performed on the same day within 3 h of each other. Exclusion criteria included previous spine surgery, neuromuscular and congenital scoliosis, metabolic diseases affecting the spine, and contraindications to MRI examination.

### Radiographic techniques and magnetic resonance imaging acquisition

X-ray examinations were performed on an Axiom Aristos FX system (Siemens, Erlangen, Germany) with age-adjusted settings for tube voltage and current in PA view and lateral orientation. A scatter grid was used for body diameters > 15 cm. The selected field of view included the entire spine and the iliac crest. Images were stitched before analysis. All radiographs were taken while the patient was standing without a brace.

**Fig. 2:**
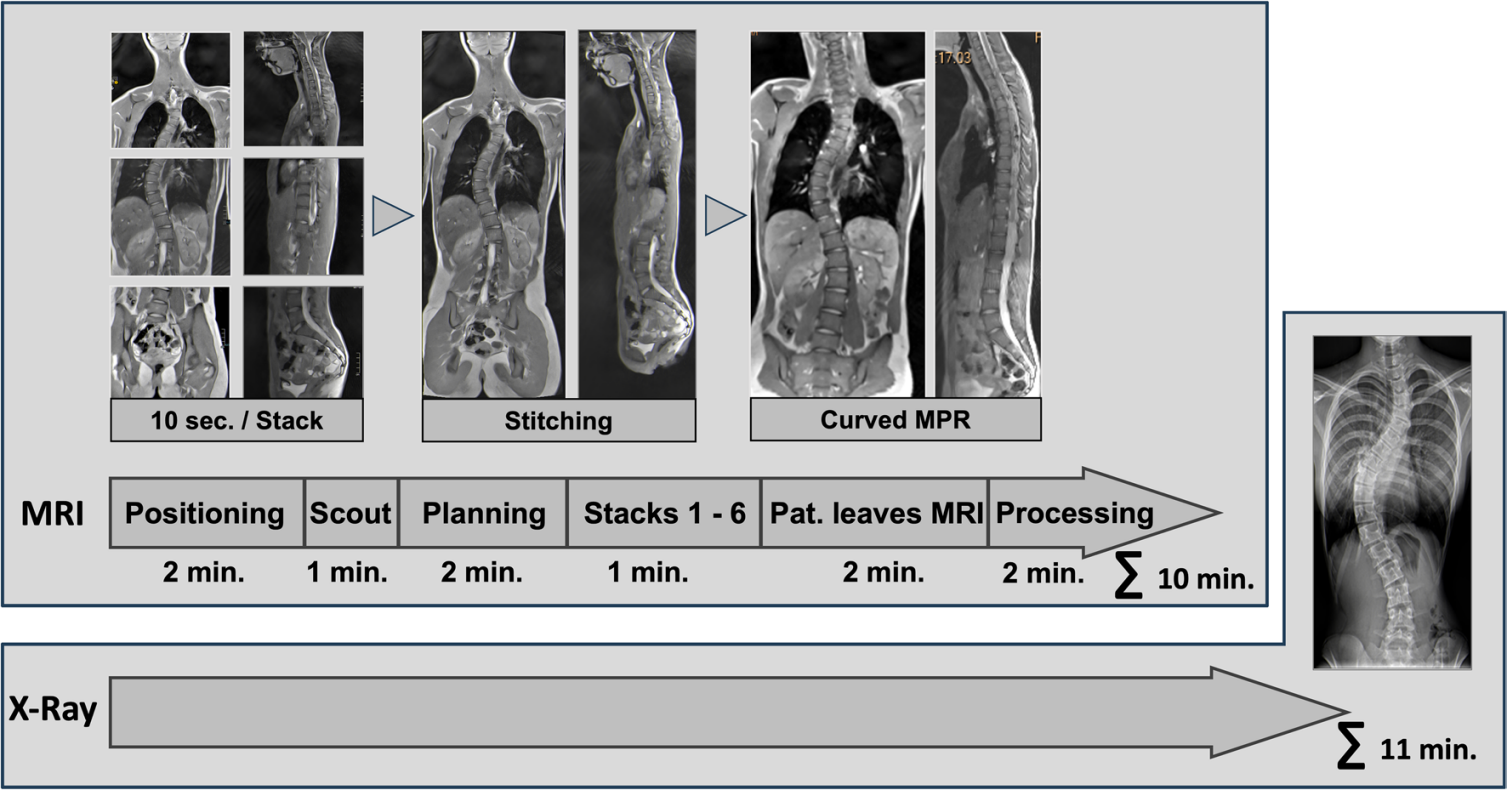
Time requirements and examination process of magnetic resonance imaging (MRI) compared to X-ray in a 16-year-old girl with a history of scoliosis. The workflow and approximate time required from entering to leaving the examination room are shown. The X-ray examination is performed with three exposures each in posteroanterior and lateral planes. The real-time MRI is shown to be faster than X-ray examination. *MPR*, multiplanar reconstruction

All MRI examinations were performed on a 3-tesla MRI scanner (Prisma fit, Siemens, Erlangen, Germany) with the patient in a supine position. The examination utilized a 32-channel spine coil extended by neck elements of a 64-channel head coil. For planning, conventional FLASH sequences were used from the skull base to the proximal femur, followed by a rapid T2/T1-weighted volume coverage sequence in both coronal and sagittal orientations with an equal field of view. This real-time MRI sequence leverages an ultra-fast frame rate to scan a larger volume within a few seconds [17–19]. Three coronal and sagittal stacks were acquired and stitched together before analysis. Measurement parameters were based on a refocused FLASH sequence to provide steady-state free precession-type T2/T1 contrast without sensitivity to banding artefacts. The parameters included a field of view of 320 mm, matrix 320, slice thickness of 3.5 mm, 21 radial k-space spokes, flip angle of 50°, repetition time/time to echo 4.12/2.06, and bandwidth of 1040 Hz/Px. The examination procedure is shown in Fig. 2.

The institution established dedicated daily time slots in the MRI timetable to ensure rapid patient turnover without affecting slots for conventional MRI.

### Radiographic and magnetic resonance evaluation

**Fig. 3:**
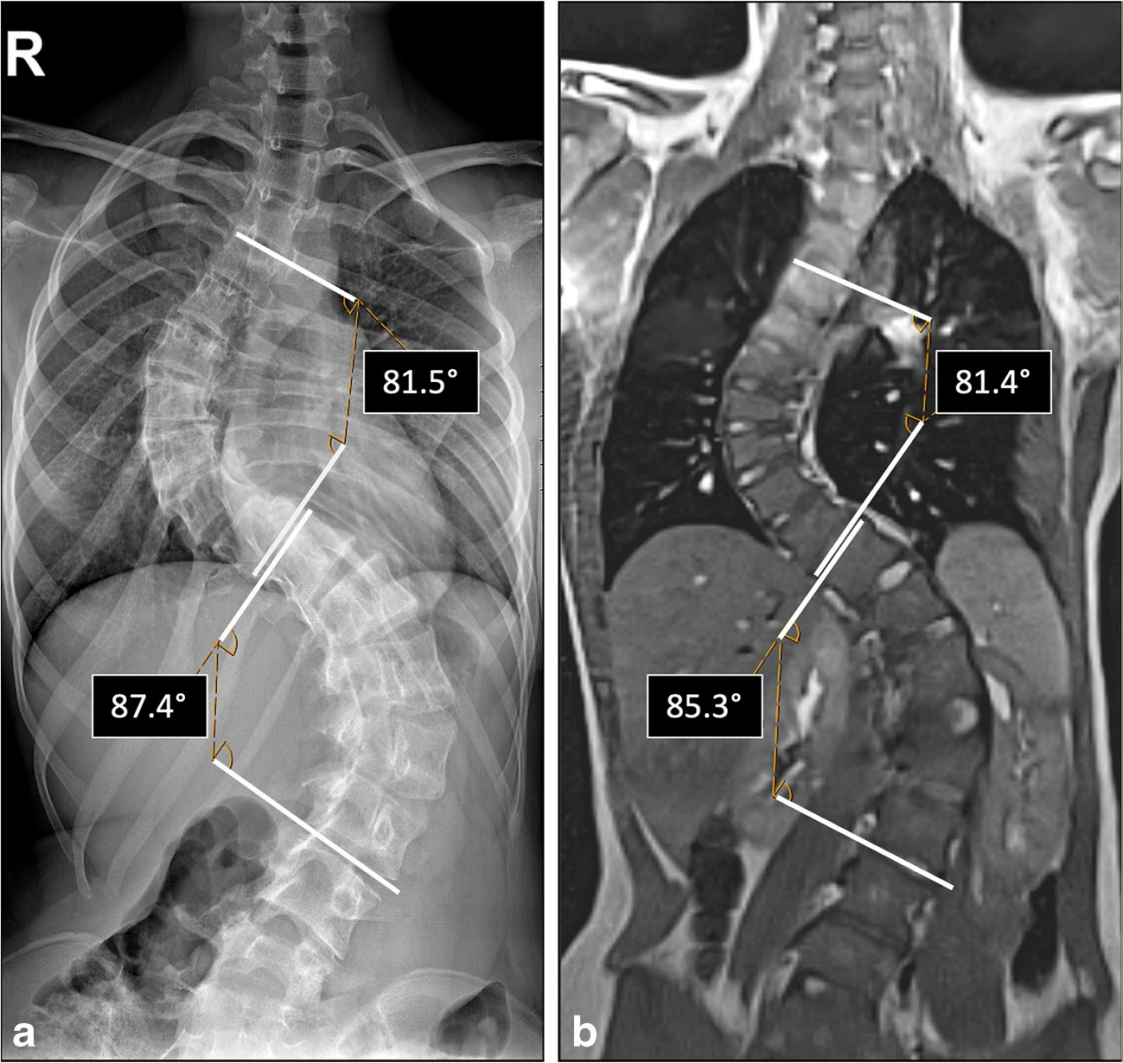
Comparison of Cobb angles on radiographs and real-time magnetic resonance imaging (MRI). A 15-year-old girl with a history of severe scoliosis. **a** Stitched whole-spine radiograph in the posteroanterior plane: S-shaped scoliosis with a right-convex thoracic curve (Cobb angle of 81°) and a left-convex thoracolumbar counter-curve (Cobb angle of 87°). **b** Stitched curved multiplanar reconstructions of the coronal real-time MRI shows corresponding Cobb angles of 81° and 85°, respectively

**Fig. 4:**
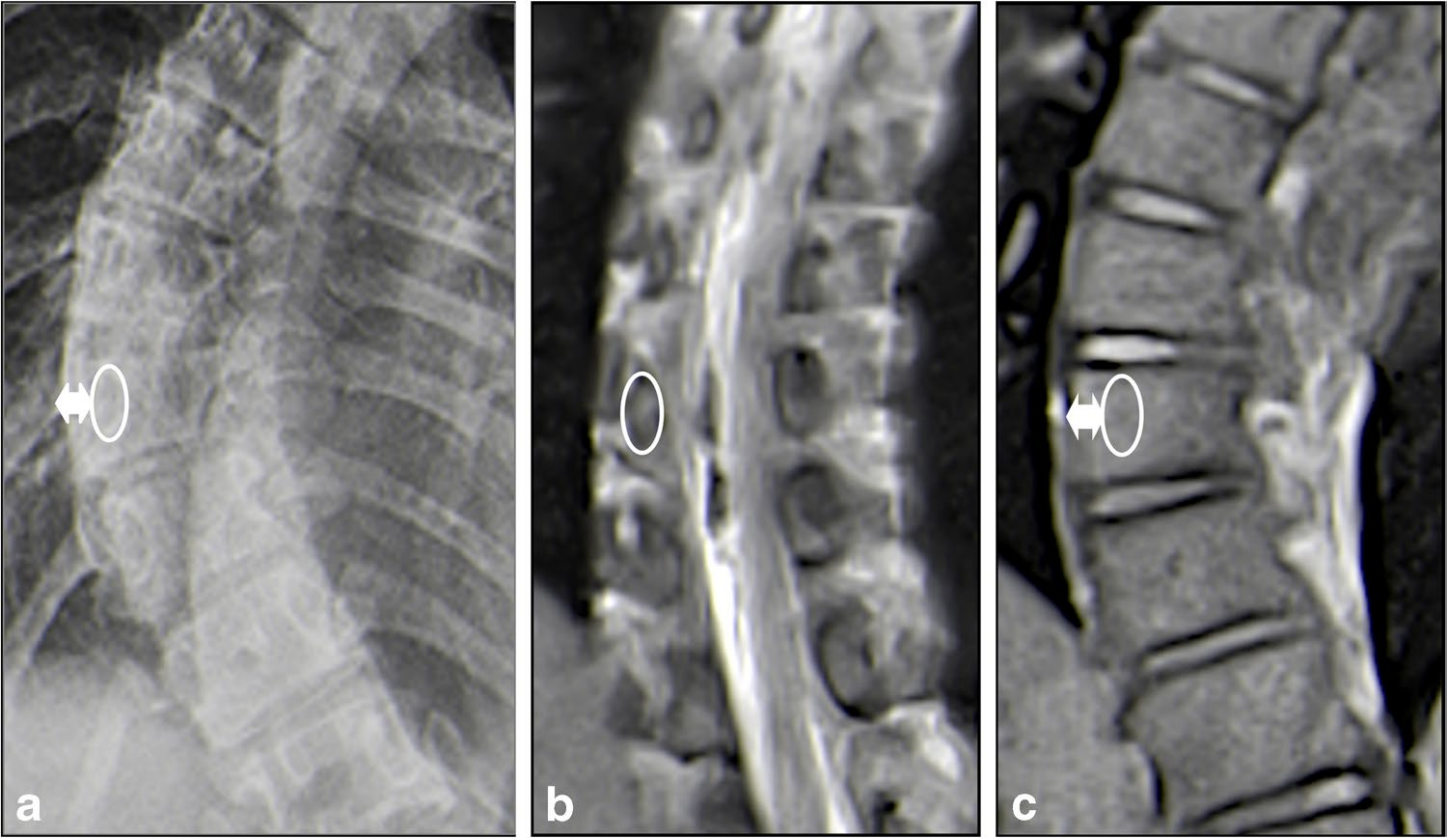
Vertebral body rotation (Nash and Moe method [4] adapted to real-time magnetic resonance imaging (MRI). **a** Evaluation of the rotation on a postero-anterior radiograph of a 12-year-old girl: measuring the distance (*double-headed arrow*) between the pedicle (*oval*) and the lateral vertebral boundary. **b**,**c** Corresponding rotation measurement on the coronal real-time MRI via cross-layer distance measurement (*double-headed arrow*) between the pedicle (*oval*) (**b**) and lateral vertebral boundary (*oval*) (**c**)

For conventional radiographs, the Cobb angle was determined on a picture archiving and communication system (Siemens, syngo.plaza, Erlangen, Germany) using a four-point angle measurement [3] as shown in Fig. 3. In addition, the degree of rotation at the level of the apex vertebra [4] (Fig. 4) and the bone maturation stage at the iliac crest, were determined [5].

### For magnetic resonance imaging

The Cobb angle was measured on the coronal volume coverage slices that had been previously assembled and processed by curved multiplanar reconstruction using cross-slice four-point angle measurements [3, 20], as shown in Fig. 3.

The Nash and Moe rotation at the apex vertebra was determined in the same manner as on radiographs. However, this method faced difficulty as the shapes of the pedicles were not visible on the same slice as the boundary of the vertebral body. Therefore, cross-layer distance measurement between the convex-sided margin of the pedicle and the lateral boundary of the vertebral body was used, as illustrated in Fig. 4.

**Fig. 5:**
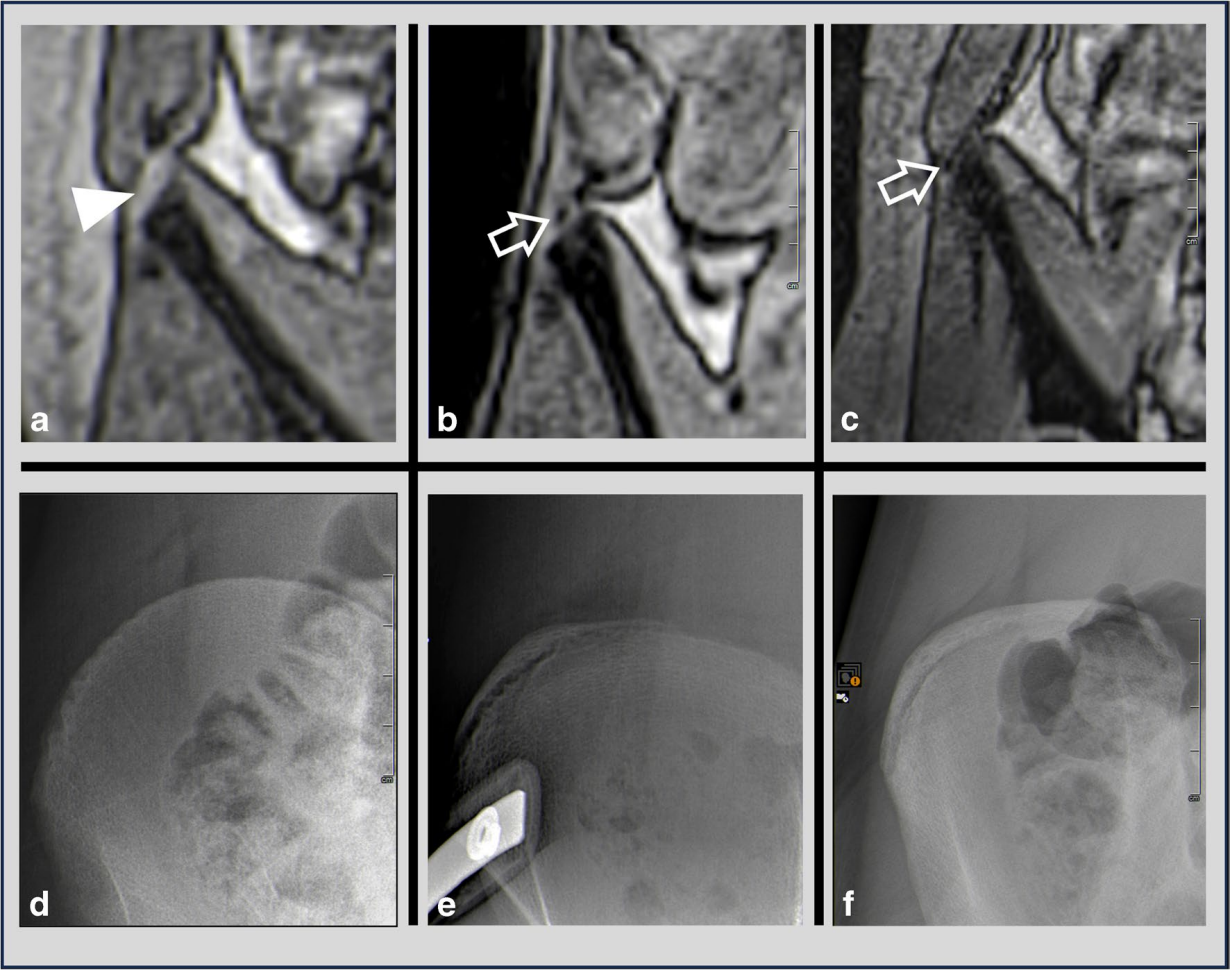
Determination of Risser stage on coronal T1-weighted magnetic resonance imaging (MRI) of the right iliac crest (**a**–**c**) and corresponding radiographs (**d**–**f**) in a 9-year-old girl (**a** and **d**), a 13-year-old girl (**b** and **e**), and a 14-year-old girl (**c** and **f**) with scoliosis. Progressive ossification of the iliac crest can be seen as a signal-free region (*arrows* in **b** and **c**) within the signal-rich cartilage (*arrowhead* in **a**). On MRI, Risser’s stage is simplified into three groups: Risser 0–1 (**d**), Risser 2–3 (**e**), and Risser 4–5 (**f**)

As the contrast between the signal-poor ossification center within the signal-rich, cartilaginous apophysis of the iliac bone is low in the volume coverage sequences, the degree of maturation according to Risser was assessed using conventional T1-weighted FLASH 3D sequences, which were required for planning (Fig. 5). To ensure everyday practicality, stages 0 (apophysis completely cartilaginous) and 1 (ossification center only ventrolateral), 2 (bone core length to approximately one-third of apophysis length) and 3 (bone core length to approximately two-thirds of apophysis length), and 4 (bone core entire apophysis length, not fused) and 5 (bone core fused to the body of the iliac bone) were combined into three groups.

Two readers independently performed the evaluation: a pediatric radiologist with 16 years of professional experience (C.R.) and a study nurse (I.K.) with 5 years of experience, who underwent a 2-month training period. The radiologist's measurements were used for further calculations.

### Statistics

The analysis was conducted using SPSS 29 (IBM Corp., Armonk, NY). Different transformation models were applied to correlate the metric-scaled value of the Cobb angles between radiographs and MRI. The decision to select the best model was based on the Pearson correlation coefficient and visually by plotting the residuals. The correlation between the ordinally scaled parameters of rotation and Risser stage on radiographs and MRI were assessed using the Spearman-Rho test and Cohen’s kappa, respectively.

The agreement among readers regarding the Cobb angle was evaluated using the intraclass correlation coefficient and graded based on Koo et al.’s criteria [21]. If a curvature was present in only one of the two modalities, the corresponding angle of the other modality was set to 0.

For the ordinal parameters of rotation and Risser stage, reader agreement was assessed using Cohen’s Kappa [22]. The significance level was set at *P*≤0.05.

## Results

Thirty-eight patients were recruited to the study. Five children were excluded: two children with spastic infantile cerebral palsy, one with neuromuscular disease, and two with vertebral body malformations. Thus, 33 children and adolescents (21 girls) with a total of 56 curvatures were included (Table 1). Seventeen subjects (51%) had S-shaped curves (corresponding to Lenke type 3), six subjects (18%) had right-convex, and five subjects (15%) had left-convex thoracolumbar scoliosis [23]. The remaining subjects were distributed as right convex-thoracic, left convex-thoracic, and reverse S-shaped. All measurements are provided in Table 2.

**Table 1:** Demographic data of the 33 patients

	Mean	Median	Standard deviation	Minimum	Maximum
Age (years)	13.7	14	± 2.3	8	17
Weight (kg)	51.7	53	± 12.0	28	91
Height (cm)	158.6	161	± 11.3	122	175

**Table 2:**
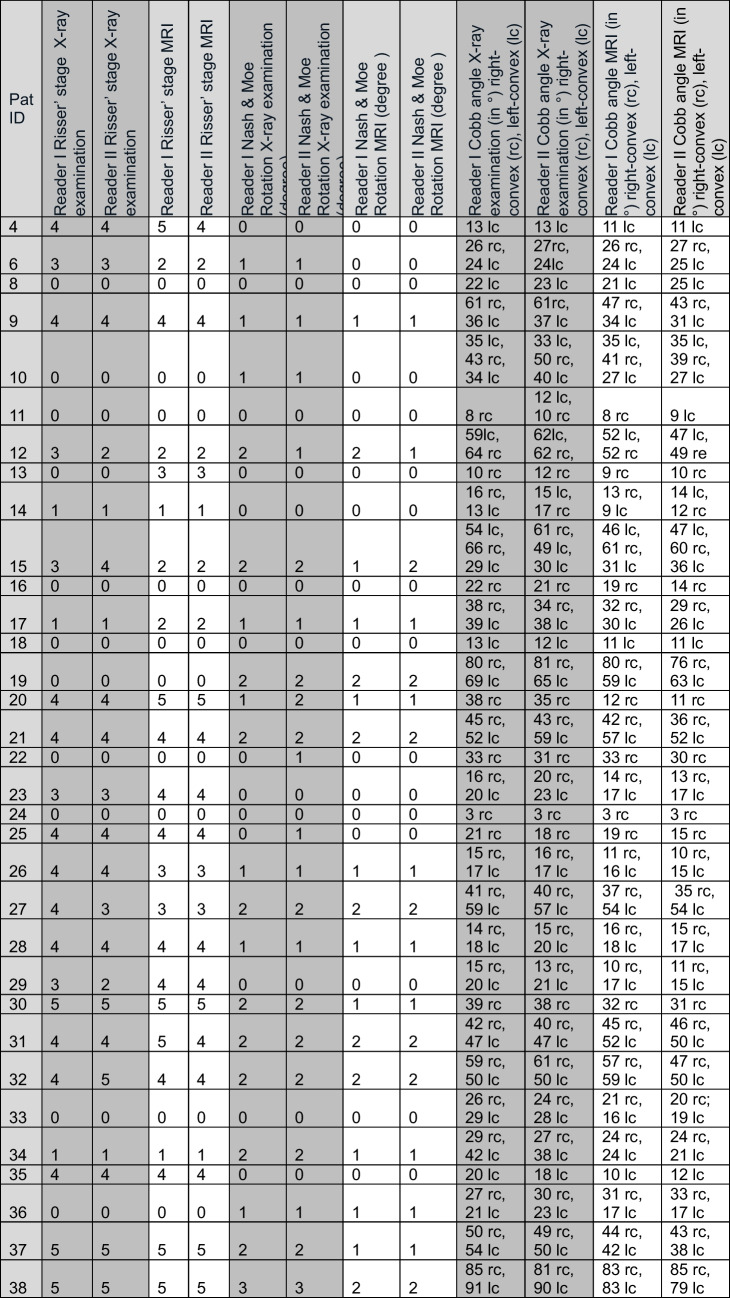
Measurement results for Cobb angle [3], rotation (according to Nash and Moe [4] and Risser stage [5] for two observers

### Cobb angle

**Fig. 6:**
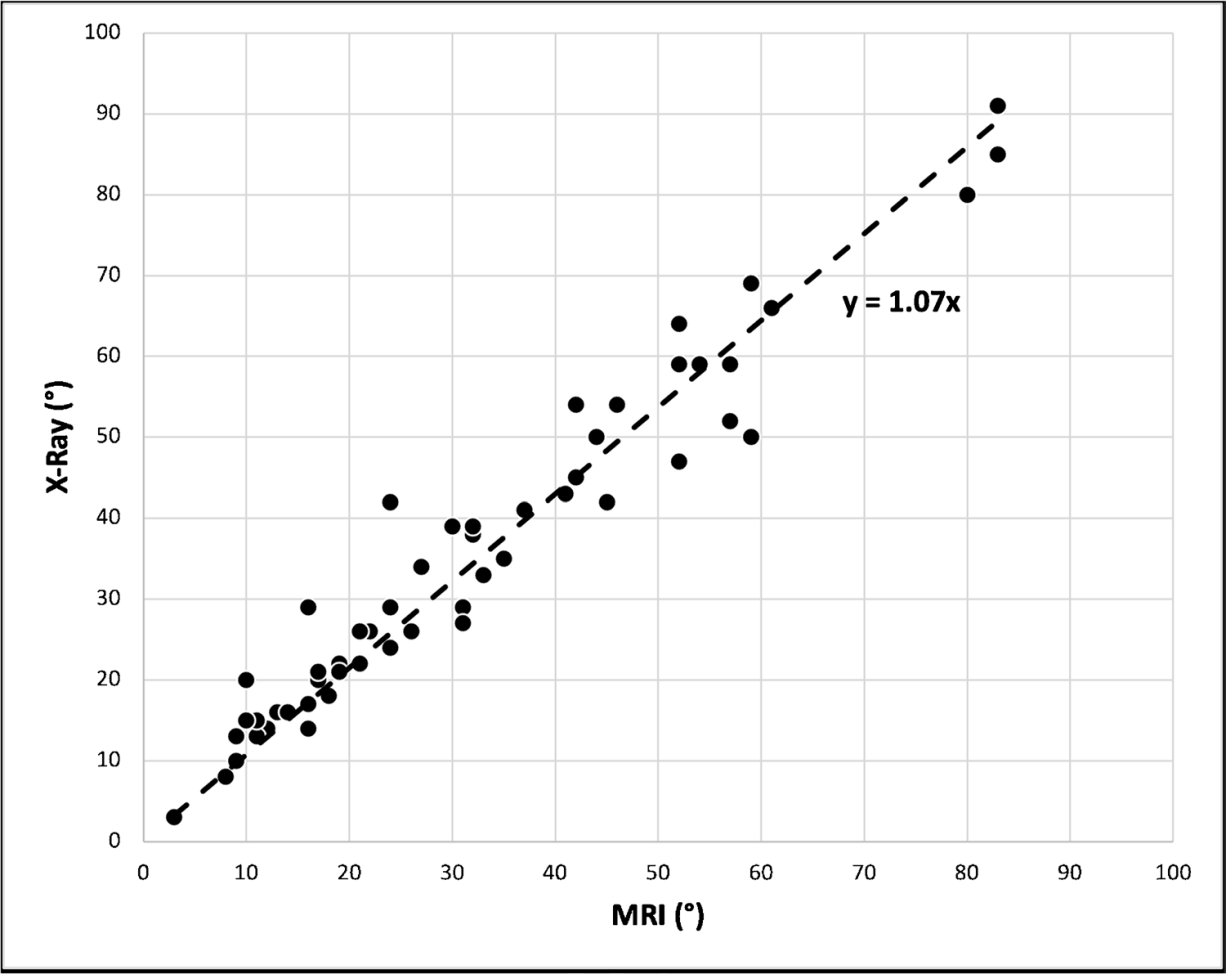
Correlation of Cobb angles measured on radiographs and real-time magnetic resonance imaging (MRI). A linear, positive correlation of the angle between the MRI and radiographic measurements was found (*R*^2^ = 0.972, *P* < 0.01). The conversion equation is Cobb_X-ray_ = 1.07*Cobb_MRI_

**Fig. 7:**
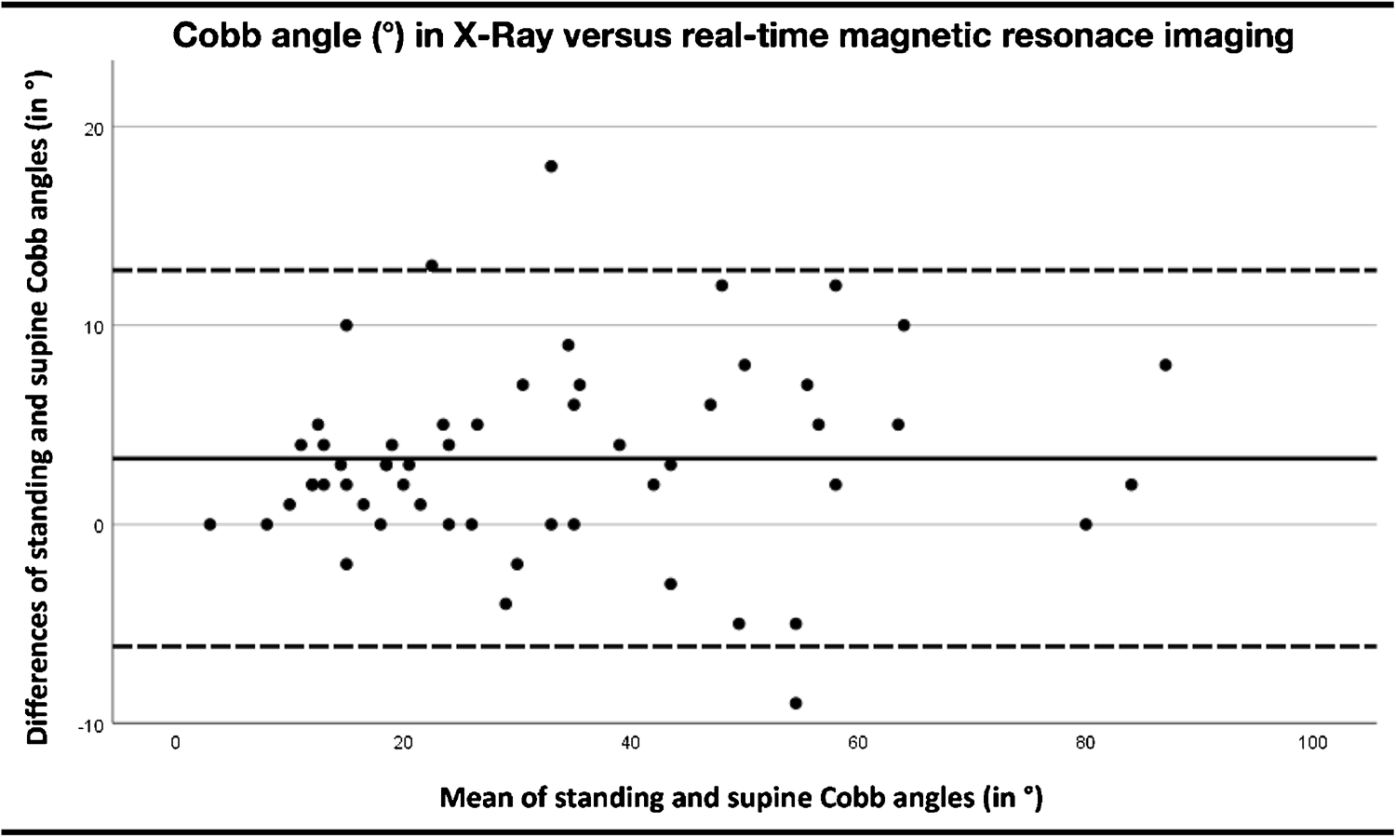
Comparison of Cobb angles in standing and supine positions. The mean of the differences in Cobb angles between standing radiographs and supine position real-time magnetic resonance imaging was 3.3°(*line*), while the standard deviation of the differences was ± 9.4° (*broken lines*)

The Cobb angle measurements between MRI and radiographs showed a linear, positive correlation (*R*^2^=0.972, *P*<0.01; Figs. 6 and 7), leading to the following conversion equation for the Cobb angle between MRI and radiography: CobbX-ray = 1.07*CobbMRI.

The interobserver variabilities were 0.99 (confidence interval [CI] 0.87–0.99) for radiography and 0.99 (CI 0.98–0.99) for MRI. The intraobserver variabilities were 0.99 (CI 0.98–0.99) for radiography and 0.99 (CI 0.96–0.99) for MRI.

### Rotation according to Nash and Moe

A linear, positive correlation was noted between the graduation obtained in the standing position on radiographs and in the supine position on MRI for the Spearman-Rho test (*R*^2^=0.92, *P*< 0.01). Rotation was graded lower on MRI than on radiography; the Cohen’s Kappa test also manifested a significant correlation (*P*<0.01) with a substantial agreement (*κ* = 0.67; Table 3) according to Landis and Koch’s classification [22]. Interobserver variability was calculated using Cohen’s kappa, yielding *κ *= 0.82 (*P* < 0.01) for radiography and *κ* = 0.90 (*P* < 0.01) for MRI.

**Table 3:**
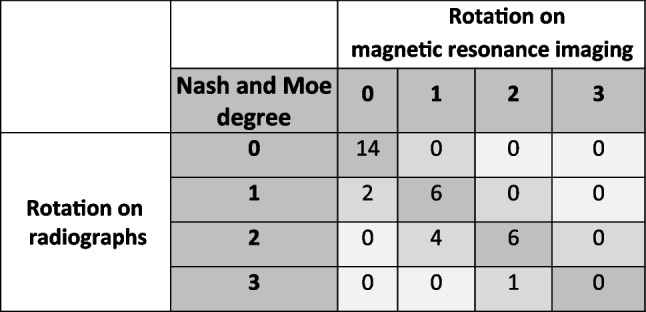
Comparison of the degrees of rotation between radiographs and real-time magnetic resonance imaging

There is a linear, positive correlation (*R*^2^ = 0.92, *P* < 0.01) between determined rotation according to Nash and Moe on radiographs and on magnetic resonance imaging (MRI). Rotation on MRI tended to be estimated slightly lower than on radiographs. Cohen’s Kappa test shows significant correlation (*P* < 0.01) with substantial agreement (*κ* = 0.67)

### Risser stage

The Spearman-Rho test revealed a strong, positive linear correlation (*R*^2^ = 0.93, *P* < 0.01) between radiographs and MRIs, with Risser’s stage (above stage 2) usually estimated to be one stage higher on MRI than on radiographs (Table 4), resulting in a significant correlation (*P* < 0.01) for Cohen’s Kappa. Due to the systematic deviation, a substantial agreement (*κ* = 0.73) was observed according to Landis and Koch’s classification [22]. Interobserver variability was *κ* = 0.80 (*P* < 0.01) for radiography and *κ *= 0.92 (*P* < 0.01) for MRI.

**Table 4:**
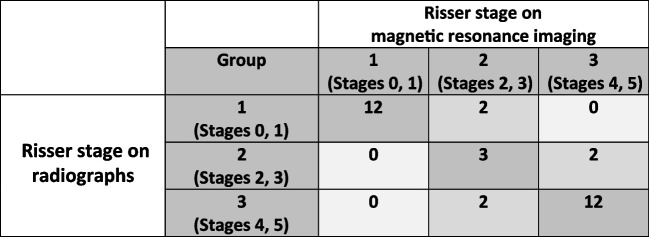
Comparison of the Risser stage between radiographs and T1-weighted magnetic resonance images

There is a linear, positive correlation (*R*^2^ = 0.93, *P* < 0.01) between the Risser stage from radiographs and from  magnetic resonance imaging (MRI). As the Risser stage above 2 was usually assessed one stage higher on MRI than on radiographs, there is also a significant correlation (*P* < 0.01) in Cohen’s Kappa. The agreement (*κ* = 0.73) is substantial

### Time requirements for radiographs and magnetic resonance imaging

The real-time MRI took a mean of 8.8 min (minimum 7.5 min, maximum 11 min, and standard deviation [SD] 1.1 min) for the whole examination; this includes the time from leaving the locker room to entering the MR room, positioning, planning, and performing the examination until leaving the MRI. As postprocessing was already included in the X-ray examination, an additional 2 min was allocated for the MRI (for stitching and curve reformations). The radiographic examination took a mean of 11.5 min (minimum 9 min, maximum 15 min, and SD 1.5 min) for both planes with three images each after leaving the locker room. This time included positioning on the wall stand, stitching, and preparation of the composite radiographs.

## Discussion

In this study, the use of real-time MRI of the spine in coronal and sagittal planes has, for the first time, been demonstrated. Our results indicate that an MRI in the supine position is an excellent alternative to a standing radiograph for determining the Cobb angle. Although conventional MRI has been evaluated as an alternative in previous studies, it has a long examination time ranging from 20 min [15] to 60 min for a complex multi-sequence examination [13]. This interval increases costs and is poorly tolerated by younger children, rendering conventional MRI less suitable for controlled scoliosis examinations).

The speed of real-time MRI has two decisive advantages: first, it is resistant to macro-movements as the acquisition time per image is barely 90 ms [17]. This distinguishes the real-time MRI sequences from, for example, the commercially available half-Fourier-acquisition single-shot turbo spin echo (HASTE) sequence. Second, the fast real-time MRI sequences are even shorter than conventional X-ray.

The scan time for the spine in coronal and sagittal planes, including the pelvis and hip joints, was just 60 s. Only the EOS system is slightly faster at 45 s [9]. However, the EOS system is prone to motion artifacts [9]. For children who cannot stand, fast real-time MRI is also an alternative. Additionally, the acquisition costs for an X-ray machine that can only be used for whole-body images are a concern [9]. Although Rose et al. showed more than five times lower radiation exposure compared to standard X-rays of the spine, the advantages do not seem to justify the high costs [24, 25].

Regarding the Cobb angle, this work demonstrated an excellent correlation between standing X-ray and supine MRI with high inter- and intra-observer reliability. One explanation could be the high tissue contrast of the T2/T1-weighted volume coverage sequences between musculature, organs, bones, and adipose tissue. Another explanation could be the superimposition-free presentation of the cross-sectional imaging itself. This presentation facilitates image interpretation and measurements in real-time MRI, especially in cases of pronounced curvatures [8, 20]. Regarding this point, our opinion is that real-time MRI is even superior to the “gold standard” radiograph [8].

Compared to previous work, the variation in the measured Cobb angles between supine and standing position was smaller, and the correction factor was derived as Cobb_X-ray_ = 1.07*Cobb_MRI_. Previously, correction factors of 5° to 15° were published [8, 13, 26]. This discrepancy could be due to several reasons; in our study, real-time MRI was performed within 3 h after radiography. In other studies, the average time between radiography and MRI was 1–6 months [13]. Furthermore, the Cobb angle of the same curve can also increase by up to 5°, depending on the time of day [20]. Additionally, the scoliotic curvature, which is more pronounced when standing, reduces only slowly after lying down. This time delay is explained by the slow reabsorption of fluid into the intervertebral discs and the relaxation of the trunk muscle tone [27]. The interval between lying down and obtaining the scan may be too short to capture complete spine relaxation. In recent literature, there has been an ongoing discussion about the accurate determination of the Cobb angle and its relationship to patient positioning. On the one hand, an error rate of up to 10° is generally considered acceptable for radiographs [13]. Therefore, the correction factor might be omitted in most cases when the examination time is short, as under the conditions of real-time MRI. On the other hand, a recent study by Yeung et al. [26] emphasized the advantages of the EOS-system for conducting exams in a standing position under weight-bearing conditions. The study revealed differences of up to 15° in the Cobb angle measurements between upright EOS scans and computed tomography scans of the spine in the prone position. This would favor the use of the correction factor as established in this work.

The spinal rotation correlated well between MRI and radiographs. The degree of rotation was often not decreased from standing to lying, and if it was, the decrease was only to the next lower degree. An explanation could be the very short phase of lying down during the MR examinations. In young patients who still have a flexible spine, the muscles in front of the bony and ligamentous structures strive to maintain the shape of the spine [28].

Previous studies on the use of MRI have focused on measuring the Cobb angle while ignoring rotation and growth potential [8, 13, 14]. Despite some limitations in accurately determining the growth potential [20], the Risser stage can be beneficial in planning follow-up examination intervals in conjunction with other prognostic factors such as scoliosis shape, Cobb angle, and patient age. The degree of maturation of the pelvic skeleton using the Risser stage, was usually estimated to be one stage higher on MRI than on radiography. This divergence may be attributed to the fact that even minor calcifications in the ossification centers of the apophysis of the ileac crest can be clearly visualized on an MRI due to their susceptibility.

Another potential advantage of real-time MRI over radiography is the large field of view, which allows for the detection of relevant concomitant pathologies. Additionally, the contrast of T2/T1-weighted volume coverage sequences is sufficient to detect a low-lying conus medullaris or significant syringomyelia. This information might initiate further diagnostics, which is particularly important, as Tully et al. stated that one in seven adolescent neurologically asymptomatic patients with idiopathic scoliosis had a spinal cord abnormality detected on MRI [29]. This is underlined by the work of Ramadorai et al. [30], who found that the prevalence of spinal MR findings in an asymptomatic pediatric population was higher than expected. Rathjen et al. [31] showed that the magnitude of the curve at the first clinical presentation is correlated with intradural pathologies.

Finally, the significant radiation exposure [8, 13] from repeated spinal radiographs needs to be addressed. Law et al. assumed that annual whole-spine examinations from the 5th to the 30th year of life indicate that the additive lifetime cancer risk in girls is twice as high as in boys [10]. It should be mentioned that after skeletal maturity is completed, only curvatures greater than 30° need to be further monitored [32], so the results might be overestimated. Oakley et al. also addressed concerns related to radiation-induced cancer risks. Even with a large number of examinations (e.g., 40–50), a maximum calculated dose of 50 mGy would still be well below the leukemia threshold of 1,100 mGy. Additionally, the activation of DNA repair mechanisms must be considered [33]. Conventional spine radiographs are generally safe; however, intermittently replacing them with real-time MRI scans seems to be a reasonable approach.

The study has some limitations. The small number of cases limits its statistical power. This work was designed as a proof of concept to show the equivalence of measurements between radiography and real-time MRI in adolescent idiopathic scoliosis. Non-idiopathic scoliosis cases were not included. Another point to consider relates to the well-known limitations of the Risser staging system. It is utilized here because it is widely used and reliable [34]. The Sanders score, which requires an additional radiograph, was not used in this study [35]. The situation is similar to the Tanner-Whitehouse classification [34]. Additionally, economic factors might limit the use of ultra-short real-time MRI. Even though the scanning procedure is much shorter, the cost is higher than that of an X-ray device and is currently only available for Siemens MR scanners.

## Summary

Real-time MRI offers equivalent diagnostic value to conventional radiography for monitoring idiopathic scoliosis without the need for ionizing radiation. The time required for an MRI examination is slightly less than that for conventional radiography. Hence, spinal real-time MRI examination is an excellent and fast alternative to conventional radiography.

## Data Availability

The data can be obtained by contacting the corresponding author.
